# Fungal Secretome Database: Integrated platform for annotation of fungal secretomes

**DOI:** 10.1186/1471-2164-11-105

**Published:** 2010-02-11

**Authors:** Jaeyoung Choi, Jongsun Park, Donghan Kim, Kyongyong Jung, Seogchan Kang, Yong-Hwan Lee

**Affiliations:** 1 Fungal Bioinformatics Laboratory, Seoul National University, Seoul 151-921, Korea; 2 Department of Agricultural Biotechnology, Seoul National University, Seoul 151-921, Korea; 3 Center for Fungal Pathogenesis, Seoul National University, Seoul 151-921, Korea; 4 Center for Fungal Genetic Resources, Seoul National University, Seoul 151-921, Korea; 5 Center for Agricultural Biomaterials, Seoul National University, Seoul 151-921, Korea; 6 Department of Plant Pathology, The Pennsylvania State University, University Park, PA 16802, USA

## Abstract

**Background:**

Fungi secrete various proteins that have diverse functions. Prediction of secretory proteins using only one program is unsatisfactory. To enhance prediction accuracy, we constructed Fungal Secretome Database (FSD).

**Description:**

A three-layer hierarchical identification rule based on nine prediction programs was used to identify putative secretory proteins in 158 fungal/oomycete genomes (208,883 proteins, 15.21% of the total proteome). The presence of putative effectors containing known host targeting signals such as RXLX [EDQ] and RXLR was investigated, presenting the degree of bias along with the species. The FSD's user-friendly interface provides summaries of prediction results and diverse web-based analysis functions through Favorite, a personalized repository.

**Conclusions:**

The FSD can serve as an integrated platform supporting researches on secretory proteins in the fungal kingdom. All data and functions described in this study can be accessed on the FSD web site at http://fsd.snu.ac.kr/.

## Background

The "secretome" refers to the collection of proteins that contain a signal peptide and are processed via the endoplasmic reticulum and Golgi apparatus before secretion [1]. In organisms from bacteria to humans, secretory proteins are common and perform diverse functions. These functions include immune system [2], roles as neurotransmitters in the nervous system [3], roles as hormones/pheromones [4], acquisition of nutrients [5-7], building and remodeling of cell walls [8], signaling and environmental sensing [9], and competition with other organisms [10-13]. Some secretory proteins in pathogens function as effectors that manipulate and/or destroy host cells with special signatures. In *Plasmodium *and *Phytophthora *species, effectors carry the RXLX [EDQ] or RXLR motifs as host targeting signals [11-13].

With the aid of advanced genome sequencing technologies [14], the rapid increase of sequenced fungal genomes offers many opportunities to study the function and evolution of secretory proteins at the genome level [15,16]. The Comparative Fungal Genomics Platform (CFGP; http://cfgp.snu.ac.kr/) [16] now archives 235 genomes from 120 fungal/oomycete species. The accurate prediction of secretory proteins in sequenced genomes is the key to realizing such opportunities.

The widely used SignalP 3.0 program [17] detected 89.81% of the 2,512 experimentally verified sequences in SPdb [18], a database containing proteins with signal peptides. To improve the accuracy of prediction, we built a hierarchical identification pipeline based on nine prediction programs (Table 1). Through this pipeline, putative secretory proteins, including pathogen effectors, encoded by 158 fungal and oomycete genomes were identified. The Fungal Secretome Database (FSD; http://fsd.snu.ac.kr/) was established to support not only the archiving of fungal secretory proteins but also the management and use of the resulting data. The FSD also has a user-friendly web interface and offers several data analysis functions via Favorite, a personalized data repository implemented in the CFGP (http://cfgp.snu.ac.kr/)[16].

**Table 1:** List of prediction programs used in FSD

Prediction Program	Description	Ref
SignalP 3.0	A program to predict whether a protein has the signal peptidase site I or not	[17]
SigCleave	A program to predict whether a protein has signal peptides or not	[19]
SigPred	A program to predict whether a protein has signal peptides or not	[20]
RPSP	A program to predict whether a protein has signal peptides or not	[21]
TMHMM 2.0c	A program to predict whether a protein has trans-membrane helix(es) or not	[26]
TargetP 1.1b	A program to predict a site where a protein probably resides	[23]
PSort II	A program to predict a site where a protein probably resides	[22]
SecretomeP 1.0f	A program to predict whether a protein is secreted by non-classical pathways or not	[25]
predictNLS	A program to predict whether a protein has nuclear localization signal or not	[28]

## Construction and content

### Evaluation of the pipeline for predicting secretory proteins

To evaluate the capabilities of four programs SignalP 3.0 [17], SigCleave [19], SigPred [20], and RPSP [21] for predicting signal peptides, we analyzed the secretory proteins collected in SPdb [18]. SignalP 3.0 identified 89.81% of 2,512 proteins; while adding the other three programs, in combination, 87.50% of the proteins, which were not predicted by SignalP 3.0, were identified. The remaining proteins (1.31% of 2,512 proteins) were investigated by using two programs that predicted subcellular localization: PSort II [22] and TargetP 1.1b [23]. We found that 34.38% of the proteins were predicted to be extracellular proteins, increasing the coverage to 99.16%. For the 1,093 characterized fungal/oomycete secretory proteins (Table 2), the combinatory pipeline raised the prediction coverage from 75.30% to 84.17% in comparison to SignalP 3.0. In addition, 98.14% of 24,921 experimentally unverified sequences in the SPdb were predicted as secretory proteins by the pipeline, while SignalP 3.0 caught 80.22% of them as positive. To assess robustness of the pipeline with non-secretory proteins, we prepared yeast proteins localized in cytosol, endoplasmic reticulum, nucleus, or mitochondrion [24]. When the 1,955 proteins were subjected to the FSD pipeline and SignalP 3.0, the numbers of false positives were almost same (84 and 82, respectively). Together, these results suggest that this ensemble approach could compensate for some of the weaknesses of individual programs, resulting in more robust predictions. Additionally, SecretomeP 1.0f [25], which can predict non-classical secretory proteins, was integrated into the FSD.

**Table 2:** List of references and annotation results of characterized fungal secretory proteins

Title	Total Identified Proteins	Class SP	Class SP^3^	Class SL	Putative Secretome	Ref
Crucial Role of Antioxidant Proteins and Hydrolytic Enzymes in Pathogenicity of *Penicillium expansum*: Analysis Based on Proteomics Approach (Secretory)	21	5	1	0	6	[43]
Crucial Role of Antioxidant Proteins and Hydrolytic Enzymes in Pathogenicity of *Penicillium expansum*: Analysis Based on Proteomics Approach (Non-secretory)	21	1	2	0	3	[43]
The *Phanerochaete chrysosporium *secretome: Database predictions and initial mass spectrometry peptide identifications in cellulose-grown medium	49	25	5	0	30	[44]
An analysis of the *Candida albicans *genome database for soluble secreted proteins using computer-based prediction algorithms (Secretory)	46	28	19	2	49	[45]
An analysis of the *Candida albicans *genome database for soluble secreted proteins using computer-based prediction algorithms (Non-secretory)	45	0	5	1	6	[45]
The secretome of the maize pathogen *Ustilago maydis *(Without known functions)	386	352	18	10	380	[46]
The secretome of the maize pathogen *Ustilago maydis *(With known functions)	168	147	15	5	167	[46]
A Catalogue of the Effector Secretome of Plant Pathogenic Oomycetes	25	22	1	0	23	[11]
Fungal degradation of wood: initial proteomic analysis of extra cellular proteins of *Phanerochaete chrysosporium *grown on oak substrate	11	8	0	0	8	[47]
Comparative proteomics of extracellular proteins in vitro and in planta from the pathogenic fungus *Fusarium graminearum*	120	63	8	0	71	[48]
Expression analysis of extracellular proteins from *Phanerochaete chrysosporium *grown on different liquid and solid substrates	27	16	4	0	20	[49]
Dandruff-associated Malassezia genomes reveal convergent and divergent virulence traits shared with plant and human fungal pathogens	34	28	0	0	28	[50]
Adaptive Evolution Has Targeted the C-Terminal Domain of the RXLR Effectors of Plant Pathogenic Oomycetes	79	79	0	0	79	[41]
Genome, transcriptome, and secretome analysis of wood decay fungus Postia placenta supports unique mechanisms of lignocellulose conversion.	47	29	3	1	33	[51]
Host-Microbe Interactions: Shaping the Evolution of the Plant Immune Response	14	12	0	1	13	[52]

**Total**	1,093	815	81	20	916	-

**Figure 1:**
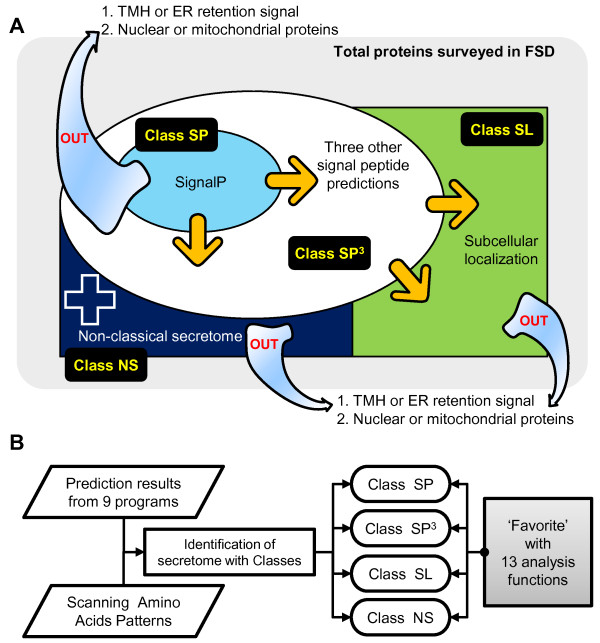
**FSD class definitions and the FSD pipeline**. (A) Definitions of four FSD classes. The gray round rectangle indicates the total set of proteins, and the light blue arrows going outside the rectangle show the filtering out processes of the pipeline. The black rectangles show the names of the classes, the yellow arrows indicate expansion of the putative secretome boundary, and the white-bordered blue cross indicates additional information on the putative secretome. (B) Structure of the FSD pipeline. The two parallelograms are input data for the FSD pipeline. The rectangle in the middle indicates the process for identifying putative secretory proteins. The round rectangles indicate the four FSD classes. The gray square on the right represents the thirteen different analysis functions in Favorite.

The FSD contains an identification pipeline that sequentially analyzes proteomes of interest using i) SignalP 3.0; ii) a combination of SigCleave, SigPred, and RPSP to screen those proteins not considered positive by SignalP 3.0; and iii) PSort II and TargetP 1.1b to analyze the negatives from the previous step. Additionally, SecretomeP 1.0f was integrated to provide information related to non-classical secretory proteins. To eliminate potential false positives, we filtered proteins that i) contain more than one transmembrane helix predicted by TMHMM 2.0c [26] and/or ii) the endoplasmic reticulum retention signal ([KRHQSA]- [DENQ]-E-L; classified as false-positive; Figure 1A) [27]. In addition, iii) nuclear proteins predicted by both predictNLS [28] and PSort II [22] and iv) mitochondrial proteins predicted by PSort II [22] as well as TargetP 1.1b [23] were eliminated because two subcellular localizations are not related to secretory proteins.

Following analysis via the pipeline, the resulting putative secretory proteins after removing potential false positives are divided into four classes: i) SP contains all proteins predicted by SignalP 3.0; ii) SP^3 ^contains the proteins predicted by SigPred, SigCleave, or RPSP but not by SignalP 3.0; iii) SL contains the proteins predicted by PSort II and/or TargetP 1.1b but not by the first two steps; and iv) NS contains the proteins predicted by SecretomeP 1.0f but not by SignalP 3.0 (Figure 1A; Table 3).

**Table 3:** Class definitions used in FSD

Class	Description*
Class SP	Proteins which are predicted by SignalP 3.0
Class SP^3^	Proteins which are predicted by SigPred, SigCleave, or RPSP
Class SL	Proteins which are predicted by PSort II or TargetP 1.1b, but are not predicted by SignalP 3.0, SigPred, SigCleave, RPSP, or SecretomeP 1.0f
Class NS	Proteins which are predicted by SecretomeP 1.0f, but are not predicted by SignalP 3.0, SigPred, SigCleave, or RPSP

* Proteins as follows were removed from all four classes described in this table: proteins which i) contain more than one trans-membrane helixes, ii) have ER retention signals, iii) predicted as mitochondrial proteins by PSort II and TargetP 1.1b, and iv) predicted as nuclear proteins by TargetP 1.1b and predictNLS.

### System structure of the FSD

To improve the expandability and flexibility of the FSD, we adopted a three-layer structure (i.e., data warehouse, analysis pipeline, and user interface) in its design. The data warehouse was established using the standardized genome warehouse managed by the CFGP (http://cfgp.snu.ac.kr/)[16] that has been used in various bioinformatics systems [15,29-35]. The pipeline layer was built with a series of Perl programs.

In addition to the prediction programs described above, ChloroP 1.1 as well as hydropathy plots [36] were included in the FSD to provide additional information on secretory proteins. Whenever new fungal genomes become available, the automated pipeline classifies them based on the predictions of nine programs, thus keeping the FSD current (Figure 1B).

MySQL 5.0.67 and PHP 5.2.9 were used to maintain database and to develop web-based user interfaces that present complex information intuitively. Web pages were serviced through Apache 2.2.11. Favorite, a personal data repository used in the CFGP (http://cfgp.snu.ac.kr/)[16], was integrated to provide thirteen functions for further analyses.

## Utility and Discussion

### Discussion

#### Secretory proteins in 158 fungal/oomycete genomes

To survey the genome-wide distribution of secretory proteins in fungi and oomycetes, we used the pipeline to analyze all predicted proteins encoded by 158 fungal/oomycete genomes. Of the 1,373,444 open reading frames (ORFs) analyzed, 92,926 (6.77%), 103,224 (7.52%), and 12,733 (0.93%) proteins belonged to classes SP, SP^3^, and SL, respectively (Table 4, 5, and 6). In total, 208,883 ORFs (15.21%) were denoted putative secretory proteins. The proteins belonging to class NS were not included in the putative secretome because they represented more than 40% of whole proteome.

**Table 4:** List and distribution of secretion-associated proteins of the fungal genomes belonging to the subphylum Pezizomycotina archived in FSD

Species	Size (Mb)	# of ORFs	Class SP	Class SP^3^	Class SL	Putative Secretome	Ref
**Fungi (Kingdom)^a^**							
**Ascomycota (Phylum)**							
**Pezizomycotina (Subphylum)**							
*Aspergillus clavatus*	27.9	9,121	754	732	81	1,567	[53,54]
*Aspergillus flavus*	36.8	12,604	1,200	990	142	2,332	[55]
*Aspergillus fumigatus *A1163	29.2	9,929	807	878	67	1,752	[54]
*Aspergillus fumigatus *AF293	29.4	9,887	781	909	84	1,774	[56]
*Aspergillus nidulans*	30.1	10,568	922	877	96	1,895	[57]
*Aspergillus niger *ATCC1015	37.2	11,200	860	883	88	1,831	N
*Aspergillus niger *CBS513.88	34.0	14,086	1,142	1,320	154	2,616	[58]
*Aspergillus oryzae*	37.1	12,063	1,060	1,064	145	2,269	[59]
*Aspergillus terreus*	29.3	10,406	934	916	81	1,931	[53]
*Botrytis cinerea*	42.7	16,448	1,163	1,287	182	2,632	N
*Chaetomium globosum*^b^	34.9	11,124	1,121	923	99	2,143	N
*Coccidioides immitis *H538.4	27.7	10,663	548	957	80	1,585	N
*Coccidioides immitis *RMSCC 2394	28.8	10,408	575	920	66	1,561	N
*Coccidioides immitis *RMSCC 3703	27.6	10,465	539	892	65	1,496	N
*Coccidioides immitis *RS	28.9	10,457	476	855	102	1,433	[60]
*Coccidioides posadasii *RMSCC 3488	28.1	9,964	546	838	95	1,479	N
*Coccidioides posadasii *Silveira	27.5	10,125	558	869	91	1,518	N
*Cochliobolus heterostrophus *C5	34.9	9,633	932	725	83	1,740	N
*Cryphonectria parasitica*	43.9	11,184	1,040	951	93	2,084	N
*Fusarium graminearum *GZ3639^c^	15.1	6,694	373	386	47	806	[61]
*Fusarium graminearum *MIPS	36.1	13,920	1,370	1,072	118	2,560	N
*Fusarium graminearum *PH-1	36.6	13,339	1,282	1,004	118	2,404	[61]
*Fusarium oxysporum*	61.4	17,608	1,613	1,297	147	3,057	N
*Fusarium solani*	51.3	15,707	1,381	1,242	155	2,778	[62]
*Fusarium verticillioides*	41.9	14,199	1,347	1,071	116	2,534	N
*Histoplasma capsulatum *G186AR	29.9	7,454	357	578	96	1,031	N
*Histoplasma capsulatum *G217B	41.3	8,038	393	583	103	1,079	N
*Histoplasma capsulatum *H143	39.0	9,547	468	842	87	1,397	N
*Histoplasma capsulatum *H88	37.9	9,445	492	832	99	1,423	N
*Histoplasma capsulatum *Nam1	33.0	9,349	398	736	79	1,213	[60]
*Magnaporthe oryzae*	41.7	11,069	1,573	833	64	2,470	[63]
*Microsporum canis*	23.3	8,777	564	702	88	1,354	N
*Microsporum gypseum*	23.3	8,876	629	669	52	1,350	N
*Mycosphaerella fijiensis*	73.4	10,327	770	778	81	1,629	N
*Mycosphaerella graminicola*	41.9	11,395	979	913	81	1,973	N
*Neosartorya fischeri*^b^	32.6	10,403	959	818	84	1,861	[54]
*Neurospora crassa*	39.2	9,842	817	788	61	1,666	[64]
*Neurospora crassa *MIPS	34.2	9,572	788	749	78	1,615	N
*Neurospora discretadiscrete*	37.3	9,948	823	800	88	1,711	N
*Neurospora tetrasperma*	37.8	10,640	849	895	73	1,817	N
*Paracoccidioides brasiliensis *Pb01	33.0	9,136	402	808	71	1,281	N
*Paracoccidioides brasiliensis *Pb03	29.1	9,264	470	823	92	1,385	N
*Paracoccidioides brasiliensis *Pb18	30.0	8,741	425	743	55	1,223	N
*Penicillium chrysogenum*	32.2	12,791	947	1,008	127	2,082	[65]
*Penicillium marneffei*	28.6	10,638	713	792	109	1,614	N
*Podospora anserina*	35.7	10,596	1,127	893	124	2,144	[66]
*Pyrenophora tritici-repentis*	38.0	12,169	1,228	912	123	2,263	N
*Sclerotinia sclerotiorum*	38.3	14,522	971	1,109	147	2,227	N
*Sporotrichum thermophile*	38.7	8,806	697	658	66	1,421	N
*Stagonospora nodorum*	37.2	15,983	1,511	1,309	142	2,962	[67]
*Talaromyces stipitatus*	35.7	13,252	748	1,116	114	1,978	N
*Thielavia terrestris*	37.0	9,815	877	855	67	1,799	N
*Trichoderma atroviride*	36.1	11,100	907	935	86	1,928	N
*Trichoderma reesei*	33.5	9,129	738	766	70	1,574	[68]
*Trichoderma virens *GV29-8	38.8	11,643	933	1,009	93	2,035	N
*Trichophyton equinum*	24.2	8,576	571	699	69	1,339	N
*Uncinocarpus reesii*	22.3	7,798	485	626	64	1,175	[60]
*Verticillium albo-atrum *VaMs. 102	32.9	10,239	1,074	815	73	1,962	N
*Verticillium dahliae *VdLs. 17	33.9	10,575	1,157	861	77	2,095	N

**Total**	2,059.4	641,257	50,164	52,111	5,578	107,853	-

^a ^Taxonomy based on [69]

^b ^Insufficient exon/intron information

^c ^Incomplete coverage of genome information

**Table 5:** List and distribution of secretion-associated proteins of the fungal genomes belonging to the subphylum Saccharomycotina and Taphrinomycotina archived in FSD

Species	Size (Mb)	# of ORFs	Class SP	Class SP^3^	Class SL	Putative Secretome	Ref
**Fungi (Kingdom)^a^**							
**Ascomycota (Phylum)**							
**Saccharomycotina (Subphylum)**							
*Candida albicans *SC5314	14.3	6,185	321	405	87	813	[70,71]
*Candida albicans *WO-1	14.5	6,160	310	385	78	773	[72]
*Candida dubliniensis*^b^	14.5	6,027	308	340	71	719	N
*Candida glabrata *CBS138	12.3	5,165	231	290	49	570	[73]
*Candida guilliermondii*	10.6	5,920	279	400	63	742	[72]
*Candida lusitaniae*	12.1	5,941	310	482	50	842	[72]
*Candida parapsilosis*	13.1	5,733	308	321	83	712	[72]
*Candida tropicalis*	14.6	6,258	360	373	76	809	[72,74]
*Debaryomyces hansenii*	12.2	6,354	254	357	74	685	[73]
*Eremothecium gossypii*	8.8	4,717	204	333	35	572	[75]
*Kluyveromyces lactis*	10.7	5,327	248	304	60	612	[73]
*Kluyveromyces polysporus*	14.7	5,367	219	276	58	553	[76]
*Kluyveromyces waltii*	10.9	4,935	187	280	41	508	[77]
*Lodderomyces elongisporus*	15.5	5,802	253	351	50	654	[72]
*Pichia stipitis*	15.4	5,839	263	374	58	695	[78]
*Saccharomyces bayanus *623-6C YM4911	11.9	4,966	200	275	44	519	[79]
*Saccharomyces bayanus *MCYC 623	11.5	9,385	663	767	141	1571	[80]
*Saccharomyces castellii*	11.4	4,677	177	240	46	463	[79]
*Saccharomyces cerevisiae *273614N	12.5	5,354	223	261	51	535	[81]
*Saccharomyces cerevisiae *322134S	12.5	5,382	224	290	53	567	[81]
*Saccharomyces cerevisiae *378604X	12.5	5,400	232	267	53	552	[81]
*Saccharomyces cerevisiae *AWRI1631	11.2	5,451	220	364	63	647	N
*Saccharomyces cerevisiae *BC187	12.5	5,332	226	263	47	536	[81]
*Saccharomyces cerevisiae *DBVPG1106	12.5	5,318	225	253	52	530	[81]
*Saccharomyces cerevisiae *DBVPG1373	12.4	5,349	229	260	48	537	[81]
*Saccharomyces cerevisiae *DBVPG1788	12.4	5,347	227	263	46	536	[81]
*Saccharomyces cerevisiae *DBVPG1853	12.5	5,359	224	265	51	540	[81]
*Saccharomyces cerevisiae *DBVPG6040	12.6	5,364	221	271	50	542	[81]
*Saccharomyces cerevisiae *DBVPG6044	12.5	5,890	224	268	48	540	[81]
*Saccharomyces cerevisiae *DBVPG6765	12.2	5,377	230	263	48	541	[81]
*Saccharomyces cerevisiae *K11	12.5	5,375	228	270	52	550	[81]
*Saccharomyces cerevisiae *L_1374	12.4	5,346	225	264	55	544	[81]
*Saccharomyces cerevisiae *L_1528	12.4	5,346	227	258	48	533	[81]
*Saccharomyces cerevisiae *M22	10.8	6,755	249	399	62	710	[82]
*Saccharomyces cerevisiae *NCYC110	12.5	5,408	226	264	57	547	[81]
*Saccharomyces cerevisiae *NCYC361	12.6	5,360	228	261	49	538	[81]
*Saccharomyces cerevisiae *RM11-1a	11.7	5,696	264	283	63	610	N
*Saccharomyces cerevisiae *S288C	12.2	6,692	394	425	99	918	[83]
*Saccharomyces cerevisiae *SK1	12.4	5,433	233	269	55	557	[81]
*Saccharomyces cerevisiae *UWOPS03_461_4	12.6	5,329	218	268	51	537	[81]
*Saccharomyces cerevisiae *UWOPS05_217_3	12.6	5,350	217	264	47	528	[81]
*Saccharomyces cerevisiae *UWOPS05_227_2	12.6	5,334	220	266	51	537	[81]
*Saccharomyces cerevisiae *UWOPS83_787_3	12.6	5,392	225	269	51	545	[81]
*Saccharomyces cerevisiae *UWOPS87_2421	12.6	5,368	226	266	56	548	[81]
*Saccharomyces cerevisiae *W303	12.4	5,467	237	271	52	560	[81]
*Saccharomyces cerevisiae *Y12	12.6	5,370	223	268	57	548	[81]
*Saccharomyces cerevisiae *Y55	12.3	5,415	239	262	60	561	[81]
*Saccharomyces cerevisiae *Y9	12.6	5,377	223	271	49	543	[81]
*Saccharomyces cerevisiae *YIIc17_E5	12.5	5,376	227	265	47	539	[81]
*Saccharomyces cerevisiae *YJM789	12.0	5,903	293	303	59	655	[84]
*Saccharomyces cerevisiae *YJM975	12.4	5,341	223	255	45	523	[81]
*Saccharomyces cerevisiae *YJM978	12.4	5,353	224	258	47	529	[81]
*Saccharomyces cerevisiae *YJM981	12.5	5,351	224	256	54	534	[81]
*Saccharomyces cerevisiae *YPS128	12.4	5,364	230	269	54	553	[81]
*Saccharomyces cerevisiae *YPS163	10.7	6,648	229	368	67	664	[82]
*Saccharomyces cerevisiae *YPS606	12.5	5,354	224	270	51	545	[81]
*Saccharomyces cerevisiae *YS2	12.6	5,383	221	254	50	525	[81]
*Saccharomyces cerevisiae *YS4	12.5	5,398	215	267	54	536	[81]
*Saccharomyces cerevisiae *YS9	12.6	5,373	226	265	51	542	[81]
*Saccharomyces kluyveri*	11.0	2,968	120	180	29	329	[79]
*Saccharomyces kudriavzevii*	11.2	3,768	187	195	28	410	[79]
*Saccharomyces mikatae*	11.5	9,016	575	630	154	1359	[80]
*Saccharomyces mikatae *WashU	10.8	3,100	161	154	24	339	[79]
*Saccharomyces paradoxus*	11.9	8,939	581	615	138	1334	[80]
*Yarrowia lipolytica*	20.5	6,524	409	464	75	948	[73]
**Taphrinomycotina (Subphylum)**							
*Pneumocystis carinii*^b, c^	6.3	4,020	129	333	35	497	N
*Schizosaccharomyces japonicus*	11.3	5,172	207	312	25	544	N
*Schizosaccharomyces octosporus*	11.2	4,925	190	263	26	479	N
*Schizosaccharomyces pombe*	12.6	5,058	192	288	36	516	[85]

**Total**	853.1	383,828	17,389	21,403	3,937	42,729	-

^a ^Taxonomy based on [69]

^b ^Insufficient exon/intron information

^c ^Incomplete coverage of genome information

**Table 6:** List and distribution of secretion-associated proteins of the fungal genomes belonging to the phyla Basidiomycota, Chytridiomycota, and Microsporidia, the subphylum Mucoromycotina, and the phylum Peronosporomycota (oomycetes) archived in FSD

Species	Size (Mb)	# of ORFs	Class SP	Class SP^3^	Class SL	Putative Secretome	Ref
**Fungi (Kingdom)^a^**							
**Basidiomycota (Phylum)**							
**Agricomycotina (Subphylum)**							
*Coprinus cinereus*	36.3	13,410	1,189	1,032	119	2,340	N
*Cryptococcus neoformans *Serotype A	18.9	6,980	377	549	56	982	N
*Cryptococcus neoformans *Serotype B	19.0	6,870	331	529	44	904	N
*Cryptococcus neoformans *Serotype D B-3501A	18.5	6,431	342	523	39	904	[86]
*Cryptococcus neoformans *Serotype D JEC21	19.1	6,475	344	541	38	923	[86]
*Laccaria bicolour*	64.9	20,614	1,190	2,024	256	3,470	[87]
*Moniliophthora perniciosa*	26.7	13,560	843	1,127	126	2,096	N
*Phanerochaete chrysosporium*	35.1	10,048	793	933	83	1,809	[88]
*Pleurotus ostreatus*	34.3	11,603	1,039	1,058	106	2,203	N
*Postia placenta*	90.9	17,173	1,057	1,808	202	3,067	[51]
*Schizophyllum commune*	38.5	13,181	975	1,175	119	2,269	N
**Pucciniomycotina (Subphylum)**							
*Melampsora laricis-populina*	21.9	16,694	1305	1483	233	3,021	N
*Puccinia graminis*	88.7	20,567	1,931	2,020	230	4,181	N
*Sporobolomyces roseus*	21.2	5,536	187	592	43	822	N
**Ustilaginomycotina (Subphylum)**							
*Malassezia globosa*	9.0	4,286	211	378	37	626	[50]
*Ustilago maydis *521	19.7	6,689	789	583	10	1382	[89]
*Ustilago maydis *FB1	19.3	6,950	481	717	34	1232	[89]
*Ustilago maydis *MIPS	19.7	6,787	574	687	34	1295	N
**Chytridiomycota (Phylum)**							
*Batrachochytrium dendrobatidis *JAM81	24.3	8,732	806	750	108	1,664	N
*Batrachochytrium dendrobatidis *JEL423	23.9	8,818	650	785	91	1,526	N
**Mucoromycotina (Subphylum *incertae sedis*)**							
*Mucor circinelloides*	36.6	10,930	580	623	83	1286	N
*Phycomyces blakesleeanus*	55.9	14,792	642	1,085	221	1,948	N
*Rhizopus oryzae*	46.1	17,482	750	994	202	1,946	[90]
**Microsporidia (Phylum)**							
*Antonospora locustae*^b^	6.1	2,606	166	208	62	436	N
*Encephalitozoon cuniculi*	2.5	1,996	90	135	34	259	[91]
**Alveolata (Kingdom)**							
**Apicomplexa (Phylum)**							
*Plasmodium berghei*	18.0	12,175	844	554	569	1,967	N
*Plasmodium chabaudi*	16.9	15,007	1,027	643	661	2,331	N
*Plasmodium falciparum *3D7	21.0	5,387	212	283	267	762	[92]
*Plasmodium knowlesi*	23.5	5,103	305	280	81	666	N
**Stramenopila (Kingdom)**							
**Peronosporomycota (Phylum)**							
*Hyaloperonospora parasitica*	83.6	14,789	868	1,235	132	2,235	N
*Phytophthora capsici*	107.8	17,414	1,485	1,179	136	2,800	N
*Phytophthora infestans*^b^	228.5	22,658	1,668	1,923	153	3,744	[93]
*Phytophthora ramorum*	66.7	15,743	1,670	1,372	91	3,133	[94]
*Phytophthora sojae*	86.0	19,027	2,040	1,662	96	3,798	[94]

**Total**	1,449.1	386,513	27,761	31,470	4,796	64,027	-

^a ^Taxonomy based on [69]

^b ^Insufficient exon/intron information

^c ^Incomplete coverage of genome information

**Figure 2:**
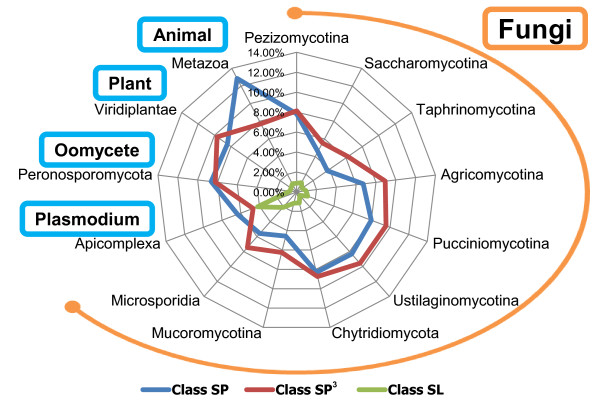
**Distribution of three classes at the phylum/subphylum level**. The average ratios of the classes to the total ORFs at the subphylum and phylum levels are described. The orange circular arc represents the fungal kingdom, and the four light blue round boxes represent phyla or kingdoms. Inside the chart, the blue line represents the ratio of class SP; the red line, class SP^3^; and the green line, class SL.

To determine the phylum-level distribution of classes SP, SP^3^, and SL within fungi, we investigated the proportions of the three classes among subphyla (Figure 2). Class SP^3 ^was the largest, class SP was a little smaller, and the class SL was much smaller; this was consistent over every subphylum. Only in *Plasmodium *species, oomycetes, and the kingdom Metazoa class SP was dominant. Class SL did not exceeded 2.10% of the whole genome, except in *Plasmodium *species (4.52%). *Plasmodium *species also showed the lowest variance among the three classes, which may reflect signal peptide-independent types of secretory proteins such as vacuolar transport signals (VTSs) [12]. These results may be partially affected by the composition of the training data for each prediction program and inherent features of each algorithm.

The phylum Basidiomycota had a larger proportion of secretory proteins (17.90%) than other fungal taxonomy such as the subphylum Mucoromycotina (11.99%) and the phyla Ascomycota (12.87%) and Microsporidia (15.10%). Within the phylum Ascomycota, the subphylum Pezizomycotina showed a higher portion of class SP (7.82%) than the subphyla Saccharomycotina and Taphrinomycotina (4.57% and 3.74%, respectively). When considered that subphylum Pezizomycotina contains many pathogenic fungi (47 of 59) compared with subphylum Saccharomycotina (11 of 65), the abundance of secretory proteins in the subphylum Pezizomycotina suggests that pathogens may have larger secretome than saprophytes in general. In fact, *Magnaporthe oryzae *and *Neurospora crassa*, a closely related pair of pathogen and non-pathogen supported by recent phylogenomic studies [37-39], contain 22.31% and 16.93% of secretory proteins, respectively. Moreover, the same tendency was found in comparison with 158 fungal/oomycete genomes archived in the FSD (pathogens and saprophytes showed 14.06% and 11.70%, respectively).

#### Effectors encoded by fungal/oomycete and *Plasmodium *genomes

*Phytophthora *species, a group that includes many important plant pathogens, uses a RXLR signal to secrete effectors to host cells [40]. RXLR effectors were tightly co-located with signal peptides predicted by the SignalP 3.0 with high confidence values (HMM and NN for 0.93 and 0.65, respectively) [41]. With the same conditions, we identified 734 putative RXLR effectors from three *Phytophthora *species, similar to a previous study [42]. However, 153 fungal genomes showed that only 0.04% of the total proteome contained this motif, suggesting that the use of RXLR for secretion is oomycete-specific.

The motivation of finding the RXLR pattern in oomycetes was the RXLX [EDQ] motif of the VTS in the malaria pathogen, *Plasmodium falciparum*. Once *P. falciparum *invades the human erythrocyte, it secretes the proteins that carry the pentameric VTS of the RXLX [EDQ] motif from the parasitophorus vacuole to the host cytoplasm [12,13]. To determine how many VTSs could be detected by our pipeline, we investigated 217 proteins of *P. falciparum *[13]. Of these, 115 proteins (53.00%) were classified as secretory proteins, defined in the FSD by the RXLX [EDQ] motif. Comparing our result to that predicted by SignalP 3.0 alone (41 out of 217), we found that our pipeline demonstrated high fidelity in detecting proteins containing VTSs.

**Figure 3:**
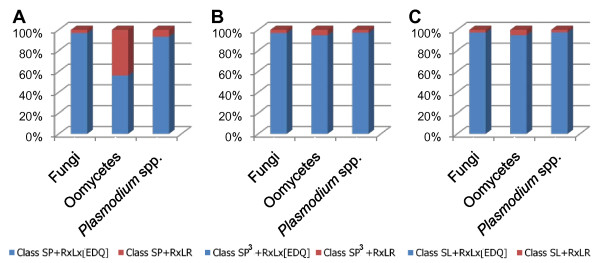
**Composition of RXLR/RXLX [EDQ] pattern in fungi, oomycetes, and *Plasmodium *species**. Composition of the RXLX [EDQ] (blue) and the RXLR (red) under class SP (A), class SP^3 ^(B), and class SL (C) with the relative ratio in fungi, oomycetes, and *Plasmodium *species, respectively.

In class SP, the proportions of proteins possessing the RXLX [EDQ] but not the RXLR motif were 96.75%, 56.18%, and 93.21% in fungi, oomycetes, and *Plasmodium *species, respectively (Figure 3A). There were similar proportions of the RXLX [EDQ] motif in classes SP^3 ^and SL across the three groups (Figure 3B and 3C). Taken together, these data show that the RXLR motif, with signal peptides predicted by SignalP 3.0, is oomycete-specific [41]. It is interesting that fungal genomes have significantly higher numbers of the RXLX [EDQ] motif than *Plasmodium *species (t-test based on amino acid frequency in each genome; *P *= 2.2e^-16^), suggesting that the RXLX [EDQ] motif may be one of fungal-specific signatures of effectors.

### Utility

#### FSD web interfaces

**Figure 4:**
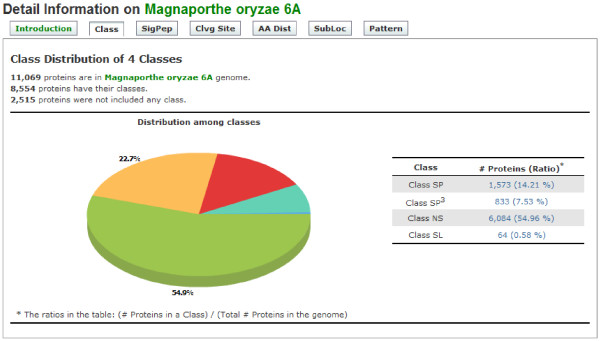
**Screenshot of genome-level analysis functions for an example fungal genome**. This screenshot shows the ORF numbers and ratios of each class through the pie chart in the left and the table in the right. The numbers in the table provide links to the list of putative secretory proteins belonging to each group. This figure shows the result from *M. oryzae*.

**Figure 5:**
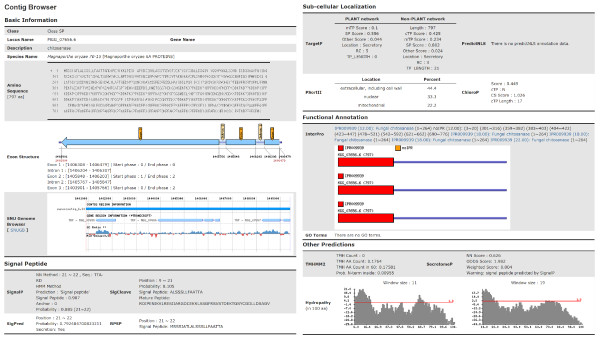
**One page summary for a protein**. The web page shows a one page summary of amino acid sequence, exon structure, and genome context via the SNUGB [15], along with 12 predictions, including signal peptides and subcellular localization.

To support the browsing of the global patterns of archived data, the FSD prepares diverse charts and tables. For example, intersections of prediction results are summarized in a chart for each genome (Figure 4). Despite of the many programs, all prediction results for each protein are displayed on one page, allowing users to browse them easily (Figure 5).

**Figure 6:**
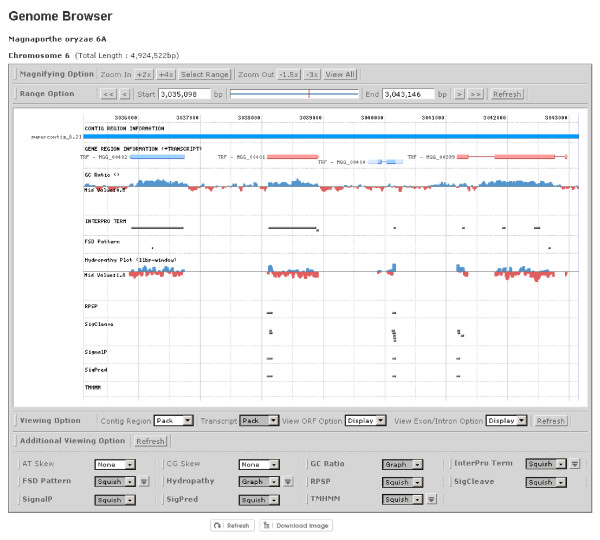
**SNU Genome Browser implemented in the FSD**. The SNUGB (http://genomebrowser.snu.ac.kr/)[15] displays i) four types of signal peptides predicted by SignalP 3.0, SigCleave, SigPred, and RPSP, ii) amino acid patterns, iii) nucleotide localization signals predicted by predictNLS, iv) transmembrane helixes predicted by TMHMM 2.0c, and v) hydropathy plots.

The SNUGB interface (http://genomebrowser.snu.ac.kr/)[15] provides several fields: i) signal peptides predicted by four different programs; ii) effector patterns, such as RXLR and RXLX [EDQ]; iii) nucleotide localization signals predicted by predictNLS; iv) transmembrane helixes predicted by TMHMM 2.0c; and v) hydropathy plots (Figure 6). The users can readily compare secretome-related information with diverse genomic contexts.

#### The personalized virtual space, Favorite, supports in-depth analyses in the FSD

**Figure 7:**
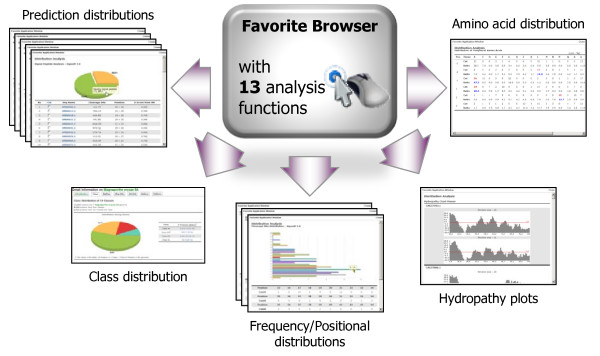
**Thirteen analysis functions in the Favorite browser**. Six different pages of analyses, connected to the Favorite browser, are displayed. "Prediction distribution" provides a list of predicted secretory proteins with their proportion to all proteins. "Class distribution" shows the composition of the classes, with the protein numbers belonging to each class. "Frequency/Position distribution" gives a bar or pie graph and numerical values linking to proteins listed for each item. "Hydropathy plots" draws the two graphs with window sizes of 11 and 19. "Amino acid distribution" presents consensus amino acids around the cleavage sites. "Functional domain distribution" lists the domains and their architecture diagrams based on InterPro terms.

The FSD allows users to collect proteins of interest and save them into the Favorite, which provides thirteen functions: i) classes distribution of proteins; ii) comparisons of predicted signal peptides generated by the four programs; iii) distributions and lists of proteins with predicted signal peptide cleavage sites; iv) compositions of amino acids near the cleavage sites; v) analyses of subcellular localization predictions; vi) lists and ratios of proteins that have chloroplast transit peptides, as determined by ChloroP 1.1; vii) analyses of proteins detected by SecretomeP 1.0f; viii) lists and distribution charts of proteins with trans-membrane helices, as predicted by TMHMM 2.0c; ix) hydropathy plots for proteins; x) analyses of proteins believed to be targeted to the nucleus of a host cell supported by predictNLS; xi) distributions and lists of proteins with a specific amino acid patterns; xii) lists of functional domains predicted by InterPro Scan; xiii) domain architecture of InterPro Scan (Figure 7). From these result pages, users can collect and store proteins in Favorite again, for further analyses. Additionally, Favorites created in the FSD can be shared with the CFGP (http://cfgp.snu.ac.kr/)[16], permitting users to use the 22 bioinformatics tools provided in the CFGP web site.

## Conclusions

Given the availability of large number of fungal genomes and diverse prediction programs for secretory proteins, a three-layer classification rule was established and implemented in a web-based database, the FSD. With the aid of an automated pipeline, the FSD classifies putative secretory proteins from 158 fungal/oomycetes genomes into four different classes, three of which are defined as the putative secretome. The proportion of fungal secretory proteins and host targeting signals varies considerably by species. It is interesting that fungal genomes have high proportions of the RXLX [EDQ] motif, characterized as host targeting signal in *Plasmodium *species. Summaries of the complex prediction results from twelve programs help users to readily access to the information provided by the FSD. Favorite, a personalized virtual space in the CFGP, serves thirteen different analysis tools for further in-depth analyses. Moreover, 22 bioinformatics tools provided by the CFGP can be utilized via the Favorite. Given these features, the FSD can serve as an integrated environment for studying secretory proteins in the fungal kingdom.

## Availability and requirements

All data and functions described in this paper can be freely accessed through the FSD web site at http://fsd.snu.ac.kr/.

## Authors' contributions

JC, JP, and YHL designed this project, JC and JP constructed the database and developed the pipeline with nine prediction programs. DK generated basic data from the twelve programs and JP, JC, and DK managed genome sequences for FSD. JC developed thirteen analysis functions of FSD. JC and JP constructed web-based interfaces. JC, JP, SK, and YHL wrote the manuscript. All the authors read and confirmed the manuscript.
